# Increased Arm Swing and Rocky Surfaces Reduces Postural Control in Healthy Young Adults

**DOI:** 10.3389/fbioe.2021.645581

**Published:** 2021-12-02

**Authors:** Cezar Mezher, Tarique Siragy, Julie Nantel

**Affiliations:** School of Human Kinetics, University of Ottawa, Ottawa, ON, Canada

**Keywords:** dynamic stability, postural control, balance, arm swing, perturbation, destabilizing surfaces, virtual reality

## Abstract

Fall-induced injuries can stem from a disruption in the postural control system and place a financial burden on the healthcare system. Most gait research focused on lower extremities and neglected the contribution of arm swing, which have been shown to affect the movement of the center of mass when walking. This study evaluated the effect of arm swing on postural control and stability during regular and rocky surface walking. Fifteen healthy young adults (age = 23.4 ± 2.8) walked on these two surfaces with three arm motions (normal, held, and active) using the CAREN Extended-System (Motek Medical, Amsterdam, NL). Mean, standard deviation and maximal values of trunk linear and angular velocity were calculated in all three axes. Moreover, step length, time and width mean and coefficient of variation as well as margin of stability mean and standard deviation were calculated. Active arm swing increased trunk linear and angular velocity variability and peak values compared to normal and held arm conditions. Active arm swing also increased participants’ step length and step time, as well as the variability of margin of stability. Similarly, rocky surface walking increased trunk kinematics variability and peak values compared to regular surface walking. Furthermore, rocky surface increased the average step width while reducing the average step time. Though this surface type increased the coefficient of variation of all spatiotemporal parameters, rocky surface also led to increased margin of stability mean and variation. The spatiotemporal adaptations showed the use of “cautious” gait to mitigate the destabilizing effects of both the active arm swing and rocky surface walking and, ultimately, maintain dynamic stability.

## Introduction

Falls and fall-related injuries that require medical attention are common debilitating issues (Bergen et al., 2016) that place a financial burden on the healthcare system (Yeoh et al., 2013; Ministry of Labour Inspection Blitzes, 2021). Falls can result from a disruption in the postural control system (Hof et al., 2005), a system tasked with the maintenance of relative segmental positioning to ensure a reliable reference frame (MacKinnon and Winter, 1993; Massion, 1994; Ivanenko and Gurfinkel, 2018). Normally, the center of mass (COM) translates smoothly in a sinusoidal trajectory in the walking direction and is kept, during double support, within the base of support (BOS) by the safe placement of the foot on the ground (Winter, 1995). This dynamic between the COM and the BOS allows for a safe and efficient gait (Lugade et al., 2011), which can be evaluated using spatiotemporal parameters, linear and angular segmental kinematics to assess postural control (Schooten et al., 2011), while margin of stability (MOS; distance between the edge of the BOS and the extrapolated COM position) (Hof et al., 2005) and coefficient of variation (COV) (Siragy et al., 2020) have been shown relevant to evaluate gait dynamic stability (Siragy and Nantel, 2018). As each measure gives insight into a particular neuromuscular control component (Siragy and Nantel, 2018), it is essential to consider the objective of the study when selecting metrics to assess human locomotion.

Typically, most gait studies assessing stability and fall risk tend to focus on the lower extremities (Gates et al., 2012; Choi and Kim, 2015) and rely on the inverted pendulum model (Winter, 1995), thereby neglecting the contribution of the arms. Therefore, the potential impact of arm motion on trunk stability was neglected as it was considered a passive product of trunk motion (Winter, 1995; Meyns et al., 2013). However, electromyography studies reported an active contribution of the shoulder muscles to arm swing (Collins et al., 2009; Meyns et al., 2013), which could actively contribute to gait stability (Collins et al., 2009).

Currently, the literature remains conflicting with regards to the impact of arm motion on postural stability when walking. Arm motion during gait has been shown to aid dynamic stability by counteracting the lower body’s angular momentum (Ortega et al., 2008; Nakakubo et al., 2014; Punt et al., 2015; Angelini et al., 2018). Yet, other studies (Bruijn et al., 2010; Pijnappels et al., 2010) showed that walking with restricted arm motion improved dynamic stability through increased trunk inertia, which reduced COM displacement. Further, some examinations of active arm swing have also demonstrated a positive association between active arm swing and dynamic stability based on local divergence exponent (Hu et al., 2012; Punt et al., 2015; Wu et al., 2016). In a prior study, our group showed that active arm swing led to increased gait variability of spatiotemporal parameters and decreased stability based on the harmonic ratios (a measure of a signal’s periodicity), which stemmed from the increase in trunk kinematics variability (Siragy et al., 2020). Nonetheless, arm motion has been shown to affect the COM’s trajectory in a steady-state condition.

However, in the case of perturbations, the gait pattern is altered and becomes more variable (Madehkhaksar et al., 2018). Perturbations in the anteroposterior (AP) direction, such as adopting an asymmetric gait pattern, can impair normal COM motion, increase trunk movement, and spatiotemporal variability (Madehkhaksar et al., 2018; Siragy et al., 2020). Perturbations in the mediolateral (ML) direction, such as lateral tugging, can generate similar adaptations, but require greater active control (McAndrew et al., 2010; McAndrew et al., 2011; Madehkhaksar et al., 2018) as ML direction has been shown to be more unstable during bipedal walking (Kuo, 1999). When walking on a rocky surface, which presents perturbations in both the AP and ML directions simultaneously, spatiotemporal parameters (step length, width, and time) are altered and become more variable as a response to the deviation of the body’s COM from its intended path (McAndrew et al., 2010; McAndrew et al., 2011; Gates et al., 2012; Hawkins et al., 2017). McAndrew Young et al. (2012) and Onushko et al. (2019) showed that perturbations such as continuous pseudo-random oscillations in the AP and ML directions led to increased foot placement variability, and sometimes, negative MOS_ML_ (an indicator of loss of stability where the extrapolated COM is outside of the BOS in the ML direction). As arm motion has been shown to affect COM motion, it is then possible that the previously mentioned arm swing strategies (normal, held, and active) can mitigate the mechanical destabilization caused by this challenging terrain and reduce the gait variability (Wu et al., 2016).

Therefore, the present study assessed the effect of normal arm swing, held arm swing and active arm swing on postural control and dynamic stability when walking on regular and rocky surface. To our knowledge, no studies investigated the impact of arm motion in terrain inducing perturbation in both the AP and ML directions. We hypothesized that active arm swing will have a negative impact on postural control and gait dynamics on a regular surface, while rocky surface walking will decrease stability and increase spatiotemporal variability. Moreover, we expected to see an interaction between arm swing and surface type. We hypothesized that postural control and stability will be increased with normal and active arm swing while walking on a rocky surface compared walking without arm swing.

## Materials and Methods

### Participants

A convenience sample of fifteen healthy young adults from the University of Ottawa community (eight males, seven females; mean age 23.4 ± 2.8 years; mean height 170.2 ± 8.1 cm; mean weight 72.3 ± 13.5 kg) were recruited to participate in this study. Exclusion criteria were any physical discomfort using a virtual reality system, any reported injuries and/or orthopedic surgeries in the previous 12 months that could interfere with gait. All participants provided informed written consent and the study was approved by the University of Ottawa’s Institutional Review Board and the Ottawa Hospital Research Ethics Board.

### Experimental Protocol

**FIGURE 1:**
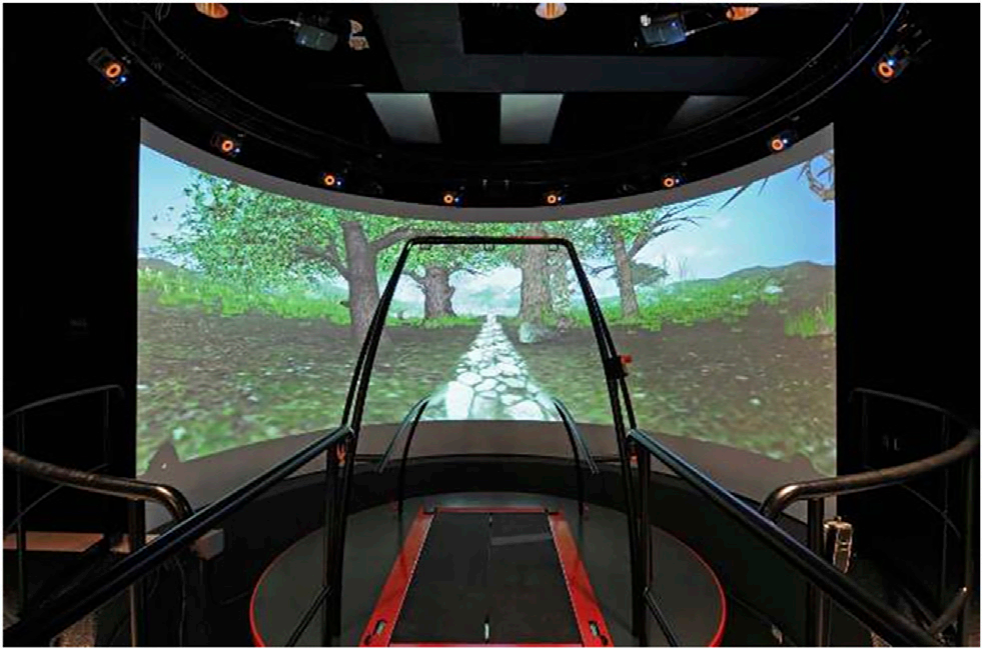
Represents the virtual park scenario within the CAREN-Extended System (Motek Medical, Amsterdam, NL). The system includes an instrumented dual-belt treadmill as well as 12 Vicon cameras.

**FIGURE 2:**
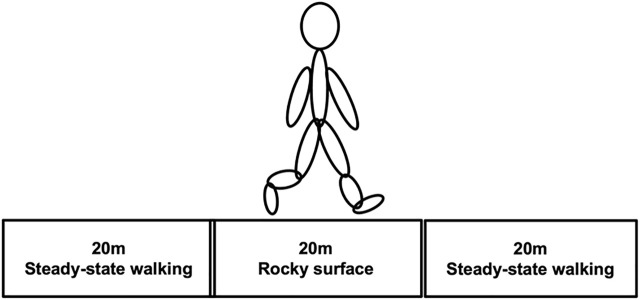
Illustrates the walking protocol.

Participants completed three-minute trials of steady-state walking at a speed of 1.2 m/s on an instrumented dual-belt treadmill using a virtual park scenario within the CAREN-Extended System (Motek Medical, Amsterdam, NL) (Figure 1). This system includes 12 Vicon cameras and an instrumented treadmill to capture kinematic and kinetic parameters. One trial was performed for each arm swing type: 1) **Normal**—participants’ natural arm motion, 2) **Held**—arms held alongside the thighs and secured in the harness, 3) **Active**—arms actively swinging to shoulder height. These were completed in a random order. Afterwards, participants were asked to walk at a self-paced speed using the same virtual park scenario. This was also repeated three times in a random order, once for each of the different arm conditions. These last walking trials consisted of steady-state walking over 20 m, followed by 20 m on the “rocky” surface and then another 20 m of steady-state walking. Using the CAREN “Rumble” module, the rocky surface was simulated through the pseudo-random oscillation of the platform in three directions simultaneously with a maximum range of ±2 cm at 0.6 Hz vertically, ±1 at 1 Hz pitch, and ±1° at 1.2 Hz roll (Sinitski et al., 2015) (Figure 2). For each trial, participants walked for 25 s to reach steady-state before beginning data collection and before the first set of 20 m steady-state walking in the rocky surface trials. Participants wore a safety harness attached to an overhead structure throughout the entire procedure. They also had the possibility to rest as necessary to minimize the effect of fatigue.

### Data Analysis

Twenty consecutive steps were taken at random from the steady-state trials to compare to 20 consecutive steps from the “rocky” terrain. The independent variables for this study were the three arm conditions (normal, held, and active arm swing) and the treadmill conditions (regular terrain and rocky surface). A baseline was established using the walking on regular surface with normal arm swing. The dependent variables for this study were trunk linear and angular velocity mean, standard deviation (SD) and maximal values. Additionally, spatiotemporal (step length, step time and step time for both legs) mean and variability were evaluated. Spatiotemporal variability was measured as the COV calculated as (SD/mean) x 100.

The MOS was calculated for both legs at respective heel-strikes. The MOS is defined as the extrapolated center of mass’s distance (xCOM) to the right/left lateral heel marker with xCOM = COM_position_ + (COM_velocity_/*ω*_θ_), where *ω*_θ_ = √*g*/*l.* In this term, *g* = 9.81 m/s^2^ and *l* is the inverted pendulum length calculated as the average distance of the right/left lateral heel marker to the COM at respective heel-strikes. The COM velocity was calculated as the first central difference of the COM’s position. The MOS was only calculated in the ML direction (Bruijn et al., 2010).

Spatiotemporal data (step length, width, time), and peak trunk angular and linear velocities for each stride were extracted and analyzed using custom scripts in Visual3D (C-Motion, Germantown, MD) and Matlab (Mathworks, Natick, MA). A 4^th^ order, low-pass Butterworth filter with a 12 Hz cut-off frequency was used to filter kinematic data.

Data was analyzed using SPSS 23.0 (IBM Analytics, Armonk, United States) and *p* < 0.05 was considered statistically significant. The Shapiro-Wilk test was used to verify normality of variables. A mixed linear model was generated to test for an interaction with arm swing and surface conditions set as fixed effects while walking speed was used as a covariate. In the event that an interaction was not found (*p*-value greater than 0.05), a test for the main effects of arm swing and walking conditions was performed. Post-hoc with a Bonferroni correction was used to compare all main effects and interactions when applicable.

## Results

Descriptive statistics for linear and angular velocities are presented in Tables 1, 2 respectively, while spatiotemporal and MOS data means and variability are reported in Tables 3, 4 respectively.

**TABLE 1:** Trunk linear velocities (x10−3 m/s) according to surface type and arm swing strategy in all three directions.

	Regular surface			Rocky surface		
	Normal	Held	Active	Normal	Held	Active
AP	4.5 ± 3.3	4.3 ± 4.8	3.7 ± 3.2	4.4 ± 2.1	3.7 ± 2.4	5.5 ± 4.1
ML*	1.6 ± 1.0	1.8 ± 1.3	1.3 ± 0.9	2.0 ± 1.4	2.9 ± 2.0	3.1 ± 2.5
V	1.9 ± 0.4	2.1 ± 0.3	1.9 ± 0.5	2.5 ± 1.1	2.3 ± 1.0	2.4 ± 1.2

Note: * shows that rocky surface led to larger values than regular surface at *p* < 0.05. AP, represents the anteroposterior direction; ML, represents the mediolateral direction and V represents the vertical direction.

**TABLE 2:** Trunk angular velocities (x10−2 °/s) according to surface type and arm swing strategy in all three directions.

	Regular surface			Rocky surface		
	Normal	Held	Active	Normal	Held	Active
AP	7.7 ± 5.7	10.2 ± 7.6	12.6 ± 9.7	15.9 ± 10.4	12.8 ± 12.0	20.6 ± 13.2
ML	12.4 ± 9.0	16.6 ± 19.4	34.9 ± 40.4	27.4 ± 22.5	41.9 ± 27.6	33.1 ± 25.8
V*	11.7 ± 6.4	12.7 ± 10.2	19.6 ± 16.9 ^‡^	12.3 ± 6.9	19.0 ± 22.3	36.34 ± 33.4 ^‡^

Note: * shows that rocky surface led to larger values than regular surface at *p* < 0.05 and ^‡^ shows that active arm swing led to larger values than normal and held at *p* < 0.05. AP, represents the anteroposterior direction; ML, represents the mediolateral direction and V represents the vertical direction.

**TABLE 3:** Average step length (cm), width (cm), time (ms) and margin of stability (cm) for both left and right heel strikes according to the surface and arm swing conditions.

		Regular surface			Rocky surface		
	Leg	Normal	Held	Active	Normal	Held	Active
Step Length	Left	56.2 ± 3.7	56.1 ± 4.0	61.3 ± 3.4^‡^	58.2 ± 9.8	53.9 ± 6.3	67.7 ± 5.8^‡^
	Right	56.6 ± 3.4	55.9 ± 3.8	60.9 ± 4.6^‡^	57.5 ± 9.8	52.0 ± 7.5	66.7 ± 7.0^‡^
Step Width	Left*	17.7 ± 3.9	17.3 ± 3.4	18.9 ± 4.0	21.4 ± 3.6	22.8 ± 5.4	22.2 ± 4.0
	Right*	17.7 ± 3.8	17.3 ± 3.5	19.0 ± 3.9	21.7 ± 3.7	22.9 ± 5.2	22.3 ± 3.8
Step Time	Left	531 ± 24	531 ± 34	577 ± 36	488 ± 49	483 ± 48	547 ± 48
	Right^#^	528 ± 27	524 ± 28	584 ± 50^‡^	488 ± 49	483 ± 48	548 ± 48^‡^
MOS	Left*	12.0 ± 1.8	12.1 ± 1.5	12.4 ± 2.2	19.9 ± 4.4	20.0 ± 5.3	20.8 ± 5.7
	Right*	10.9 ± 1.8	11.1 ± 1.9	11.3 ± 1.9	17.0 ± 2.6	18.2 ± 3.6	17.8 ± 5.4

Note: ^#^ shows that the regular surface led to larger values compared to the rocky surface at *p* < 0.01; * shows that the rocky surface led to larger values compared to the the regular surface at *p* < 0.001, while ^‡^ shows that active arm swing led to larger values than normal and held at *p* < 0.001.

**TABLE 4:** Coefficient of Variation of step length, step width and step time, as well as the standard deviation of the margin of stability (x10−2 cm) for both heel strikes according to surface type and arm swing strategy.

		Regular surface			Rocky surface		
	Leg	Normal	Held	Active	Normal	Held	Active
Step Length	Left*	1.9 ± 0.5	2.4 ± 0.8	3.0 ± 1.0	5.8 ± 1.9	7.1 ± 2.8	5.5 ± 2.2
	Right	2.4 ± 0.7	2.3 ± 0.5	3.2 ± 1.3	5.7 ± 3.4	8.1 ± 3.8	5.2 ± 3.3
Step Width	Left	8.8 ± 3.9	10.2 ± 4.6	8.7 ± 3.1	13.2 ± 3.9	12.6 ± 5.3	19.6 ± 7.0
	Right	7.9 ± 2.5	9.6 ± 4.4	9.4 ± 3.4	11.3 ± 3.9	12.8 ± 4.9	18.8 ± 6.6
Step Time	Left*	1.9 ± 0.5	2.2 ± 0.6	2.5 ± 0.8	4.8 ± 1.6	5.4 ± 1.8	4.9 ± 1.3
	Right*	1.7 ± 0.7	2.0 ± 0.6	2.5 ± 1.1	4.6 ± 2.0	5.8 ± 1.8	4.9 ± 1.4
MOS	Left	1.3 ± 0.3	1.3 ± 0.4	1.7 ± 0.6	3.9 ± 1.4	3.7 ± 1.1	5.1 ± 1.5
	Right	1.3 ± 0.4	1.2 ± 0.4	1.4 ± 0.5	3.7 ± 1.4	3.8 ± 1.3	5.6 ± 2.5

Note: * shows that rocky surface led to larger values than regular surface at *p* < 0.001.

### Sagittal Plane

**FIGURE 3:**
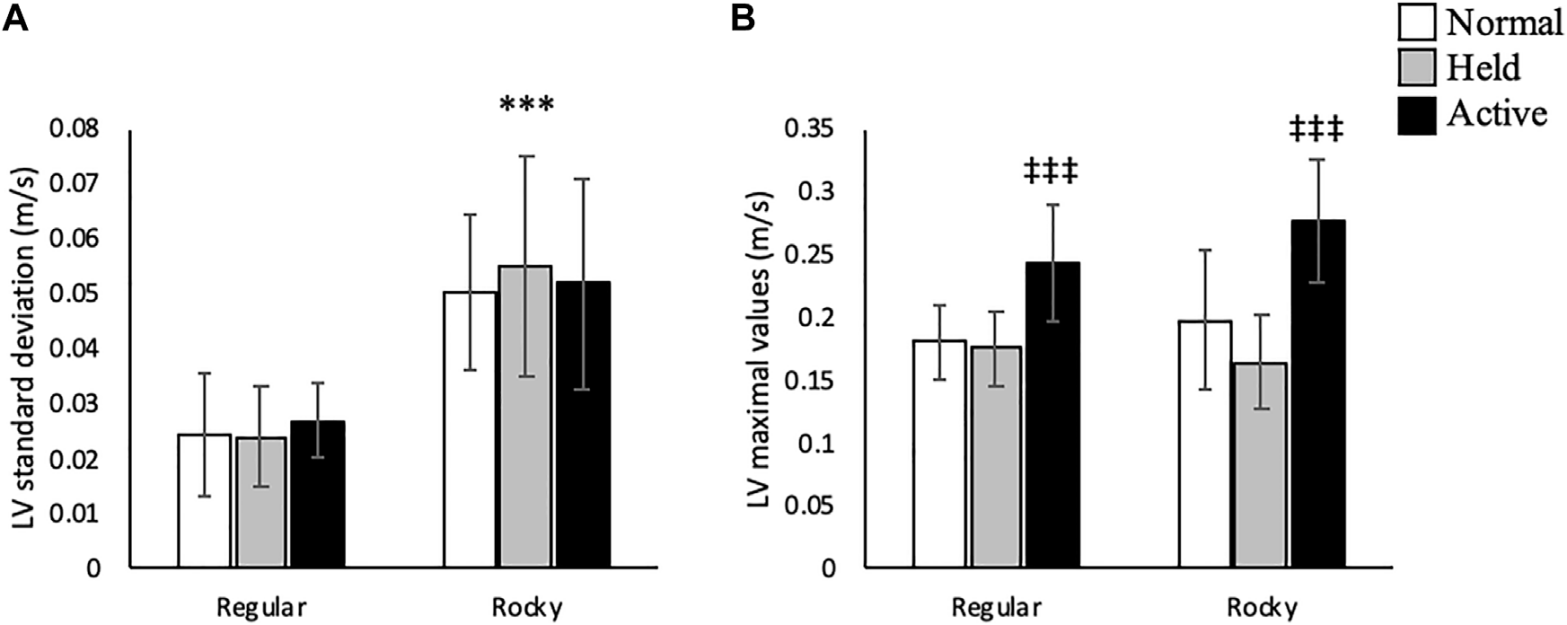
Trunk linear velocity **(A)** standard deviation (m/s) and **(B)** maximal values (m/s) according to surface type for each arm condition in the anteroposterior direction. *** shows that rocky surface led to larger values than regular surface at *p* < 0.001, while ^‡‡‡^ shows that active arm swing led to larger values than normal and held at *p* < 0.001.

**FIGURE 4:**
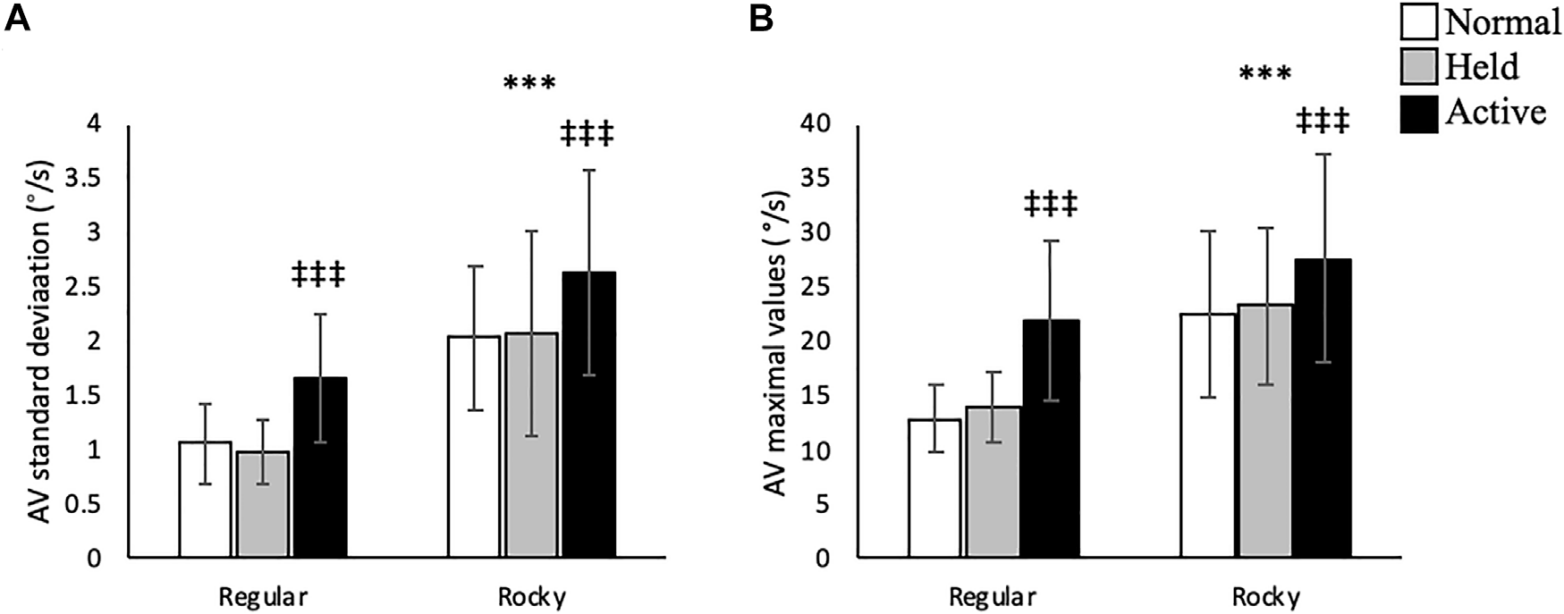
Trunk linear velocity **(A)** standard deviation (m/s) and **(B)** maximal values (m/s) according to surface type for each arm condition in the anteroposterior axis. *** shows that rocky surface led to larger values than regular surface at *p* < 0.001, while ^‡‡‡^ shows that active arm swing led to larger values than normal and held at *p* < 0.001.

In the sagittal plane, no significant interactions were detected between arm and surface conditions. A main effect for arm swing existed whereupon active arm swing increased linear velocity max [F (2, 70) = 46.66, *p* < 0.001], angular velocity SD [F (2, 70) = 16.21, *p* < 0.001] and angular velocity max [F (2, 70) = 17.36, *p* < 0.001] compared to normal and held arm swing conditions (Figures 3, 4). Additionally, a main effect for surface condition was detected, with rocky surface increasing linear velocity SD [F (1, 48) = 74.95, *p* < 0.001], angular velocity SD [F (1, 71) = 84.38, *p* < 0.001] and angular velocity max [F (1, 70) = 61.50, *p* < 0.001] compared to regular surface (Figures 3, 4).

### Frontal Plane

**FIGURE 5:**
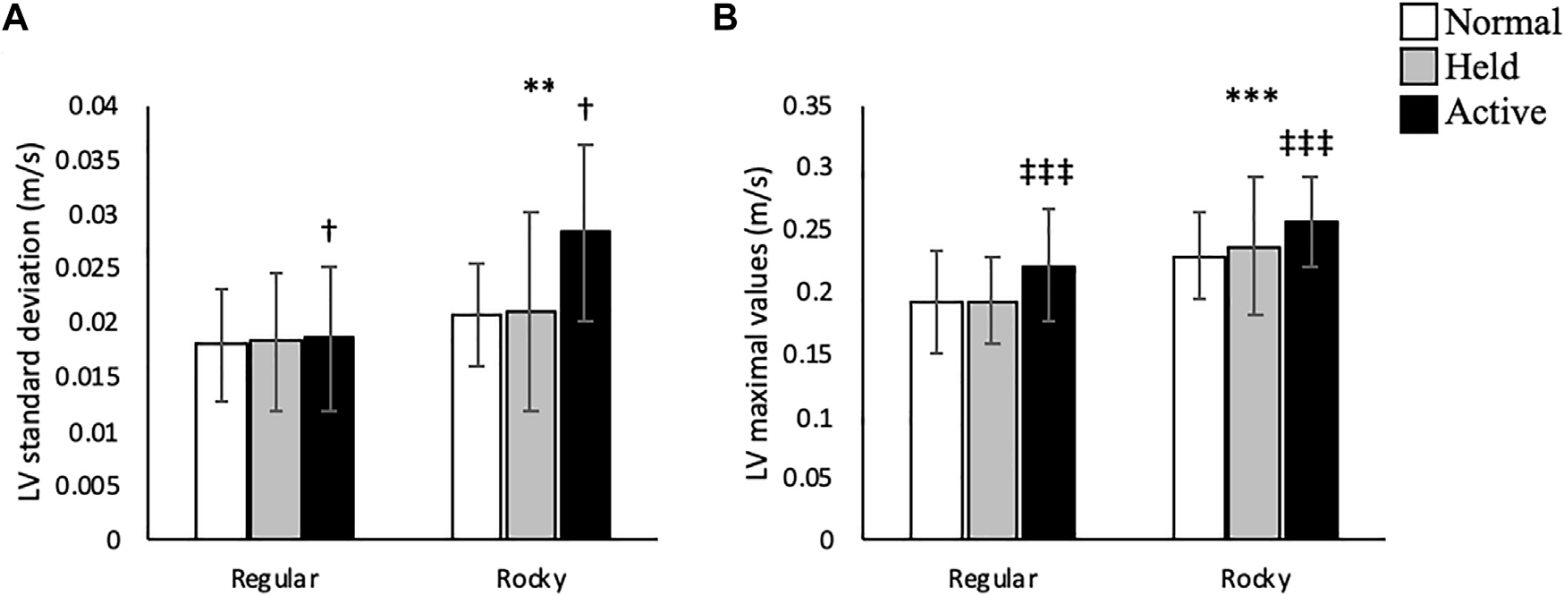
Trunk linear velocity **(A)** standard deviation (m/s) and **(B)** maximal values (m/s) according to surface type for each arm condition in the mediolateral direction. ** and *** shows that rocky surface led to larger values than regular surface at *p* < 0.01 and *p* < 0.001, while ^†^ shows that active arm swing led to larger values than normal arm swing at *p* < 0.05 and ^‡‡‡^ shows that active arm swing led to larger values than normal and held at *p* < 0.001.

**FIGURE 6:**
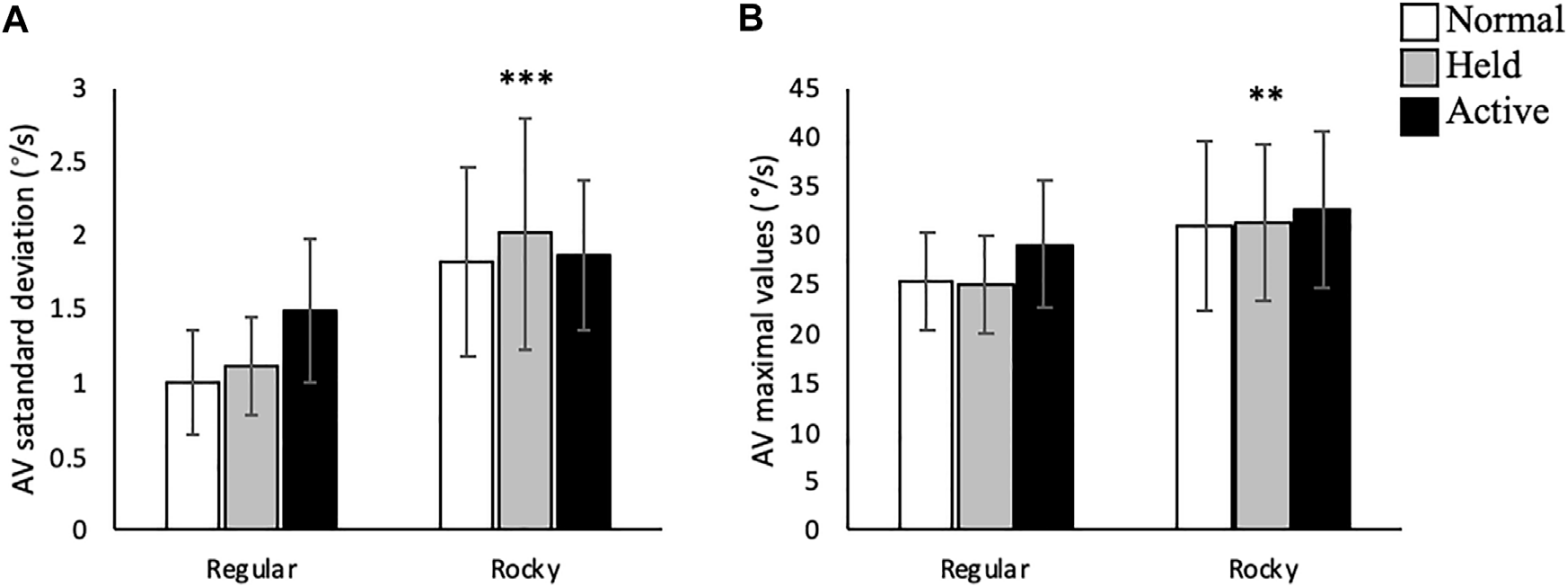
Trunk linear velocity **(A)** standard deviation (m/s) and **(B)** maximal values (m/s) according to surface type for each arm condition in the mediolateral axis. ** and *** shows that rocky surface led to larger values than regular surface at *p* < 0.01 and *p* < 0.001.

Within the frontal plane, statistical analyses revealed that active arm swing increased linear velocity SD [F (2, 68) = 4.05, *p* < 0.05] compared to normal arm swing and max linear velocity values [F (2, 70) = 8.66 *p* < 0.01] compared to both normal and held arm swing (Figure 5). A main effect was also detected for surface condition, whereupon rocky surface increased linear velocity mean [F (1, 56) = 6.20, *p* < 0.05], SD [F (1, 42) = 11.69, *p* < 0.01] and max [F (1, 70) = 42.02, *p* < 0.001] compared to regular surface (Table 1 and Figure 5). As for angular velocity, an interaction was identified for the mean [F (2, 70) = 3.25, *p* < 0.05]. The relationship between arm swing and surface conditions are shown in Figure 9A. Active arm swing led to significantly larger values compared to normal arm swing when walking on a regular surface (*p* < 0.05) and walking on a rocky surface led to larger values compared to a regular surface when using a held arm swing strategy (*p* < 0.01) (Figure 9A). Otherwise, a main effect for surface conditions was found for angular velocity SD [F (1, 70) = 49.57, *p* < 0.001] and max [F (1, 71) = 10.07, *p* < 0.01] where rocky surface led to larger values compared to regular surface (Figure 6).

### Transverse Plane

**FIGURE 7:**
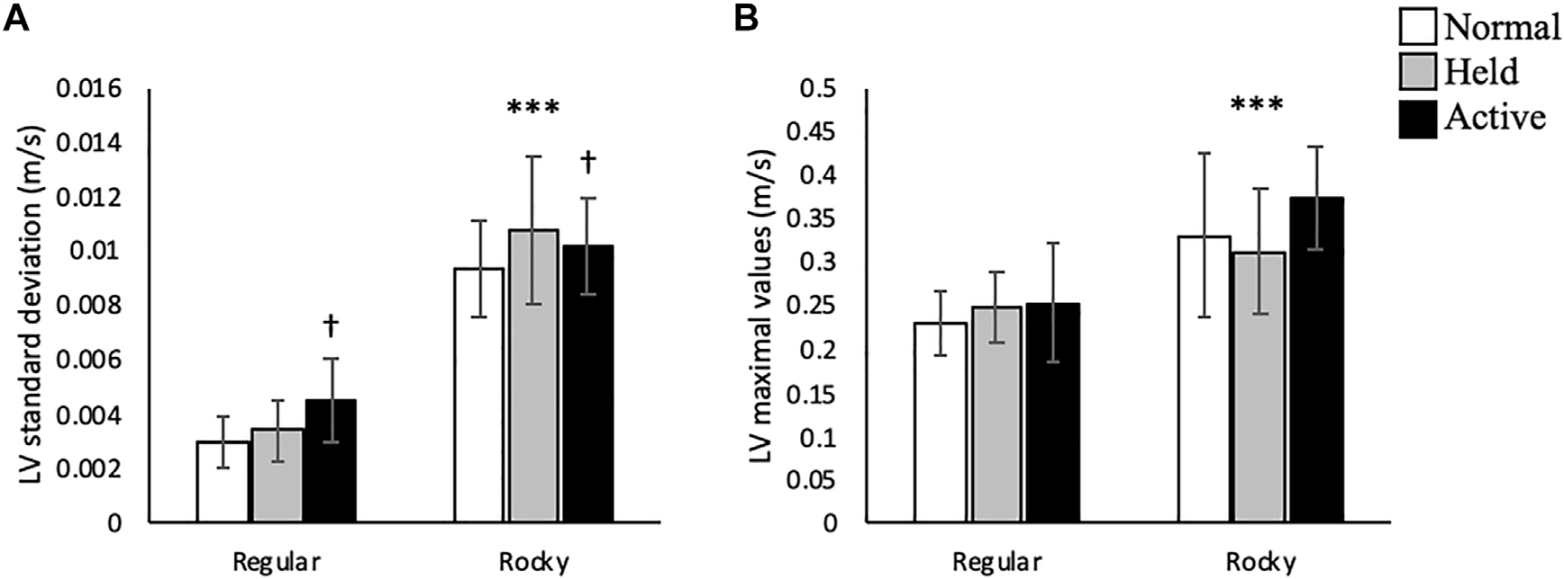
Trunk linear velocity **(A)** standard deviation (m/s) and **(B)** maximal values (m/s) according to surface type for each arm condition in the vertical direction, *** shows that rocky surface led to larger values than regular surface at *p* < 0.001 while ^†^ shows that active arm swing led to larger values than normal arm swing at *p* < 0.05.

**FIGURE 8:**
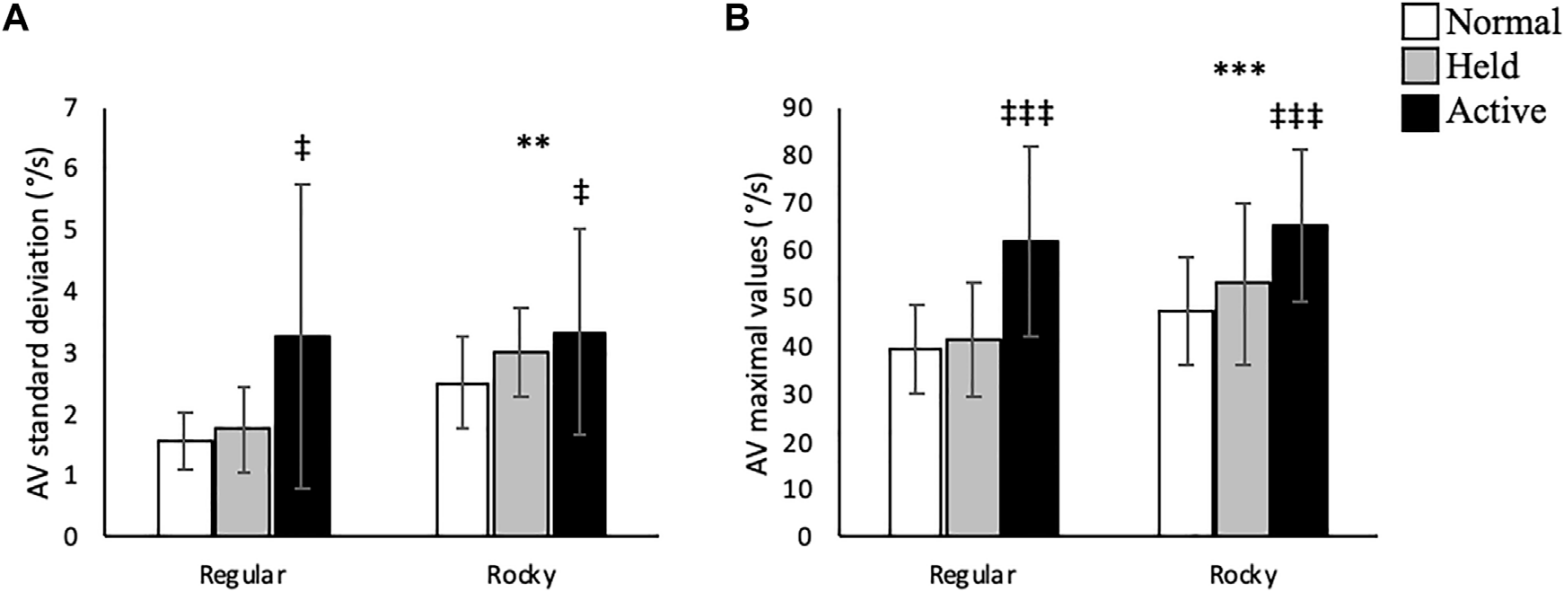
Trunk linear velocity **(A)** standard deviation (m/s) and **(B)** maximal values (m/s) according to surface type for each arm condition in the vertical direction. ** and *** shows that rocky surface led to larger values than regular surface at *p* < 0.01 and *p* < 0.001, while ^‡^ and ^‡‡‡^ shows that active arm swing led to larger values than normal and held at *p* < 0.05 and *p* < 0.001.

No interactions between arm swing and surface conditions were detected in this plane of motion. Along the vertical axis, active arm swing led to larger linear velocity SD [F (2, 70) = 4.75, *p* < 0.05] compared to normal swing (Figure 7). Moreover, active arm swing increased angular velocity mean [F (2, 70) = 6.37, *p* < 0.05], SD [F (2, 70) = 9.28, *p* < 0.05] and max [F (2, 70) = 27.86, *p* < 0.001] values when compared to normal and held arm conditions (Table 2 and Figure 8). In terms of surface condition, the rocky surface led to significantly larger values for linear velocity SD [F (1, 70) = 366.02, *p* < 0.001], linear velocity max [F (1, 72) = 40.60, *p* < 0.001], as well as angular velocity mean [F (1, 70) = 4.33, *p* < 0.05], SD [F (1, 70) = 9.06, *p* < 0.01] and max values [F (1, 70) = 10.15, *p* < 0.01] compared to the regular surface (Table 2; Figures 7, 8).

### Spatiotemporal

**FIGURE 9:**
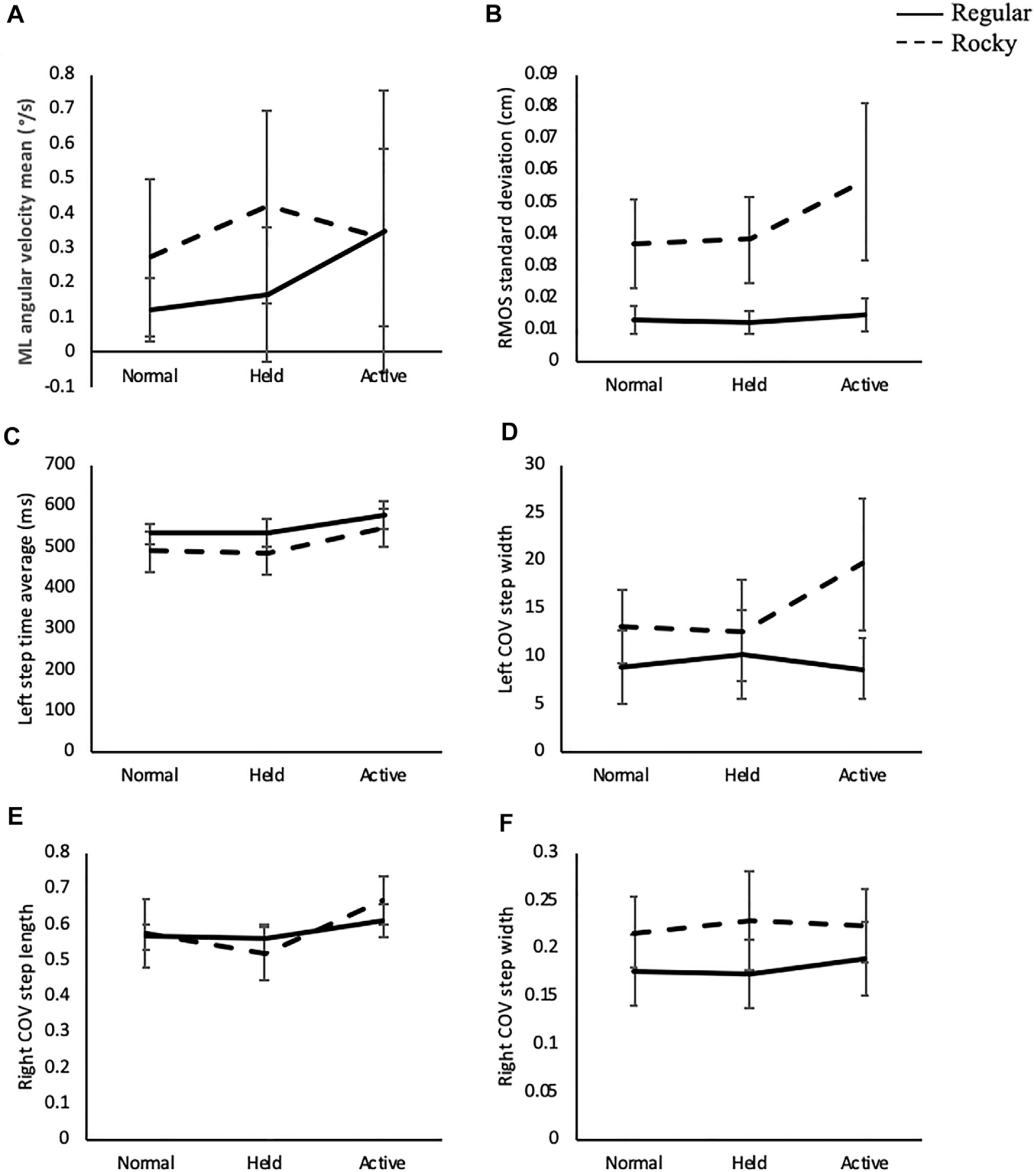
Interaction figures according surface type and arm swing strategy used for **(A)** angular velocity mean ( /s) about the mediolateral axis, **(B)** MOS standard deviation at right heel strike (cm). **(C)** step time average (ms) at left heel strike, **(D)** COV of step width at left heel strike, **(E)** COV of step length (cm) and **(F)** step width (cm) at right heel strike.

There was an interaction detected for the average step time on the left leg [F (2, 70) = 4.59, *p* < 0.05], with the relationship between the two independent variables shown in Figure 9C. Active arm swing led to significantly larger step times than normal and held arm swings (*p* < 0.001) on both regular and rocky surface (*p* < 0.001) (Table 3). In addition, a regular walking surface led to larger values compared to walking on a rocky surface (*p* < 0.001) no matter the arm swing strategy used (Table 3). Statistical analysis also revealed that active arm swing main increased the average step length of the left [F (2, 70) = 28.03, *p* < 0.001] and right [F (2, 70) = 27.67, *p* < 0.001] leg, as well as right average step time [F (2, 69) = 64.96, *p* < 0.001] compared to the normal and held arm conditions (Table 3). Furthermore, the rocky surface increased the average step width following a left heel strike [F (1, 70) = 72.34, *p* < 0.001] and a right heel strike [F (1, 70) = 74.46, *p* < 0.001], while decreasing the average step time at right heel strike [F (1, 71) = 11.11, *p* < 0.01] (Table 3).

In terms of the COV of spatiotemporal parameters, interactions were revealed for right step length [F (2, 70) = 3.54, *p* < 0.0.5] and step width at both left [F (2, 69) = 8.63, *p* < 0.001] and right [F (2, 70) = 5.70, *p* < 0.01] heel strikes. These interactions are displayed in Figures 9D–F. For the right step length, the held arm swing led to larger values compared to normal and active when walking on a rocky surface (*p* < 0.01). This challenging surface led to larger values despite the arm strategy used by the participants (*p* < 0.05) (Figure 9D). As for step width, active arm swing led to larger values compared to the other two arm swing strategies only when walking on a rocky surface at both heel strikes (*p* < 0.001) (Figures 9E,F). Also, in general, rocky surface led to significantly larger values than regular surface walking at both heel strikes no matter the arm swing used (*p* < 0.05) (Figures 9D–F). The only exception to this is the COV of step width at left heel strike when using the held arm swing; rocky surface still led to larger values, but the difference yielded a *p*-value greater than 0.05 (Table 9B in the appendix). Additionally, statistical analyses showed larger left step length variability [F (1, 75) = 142.06, *p* < 0.001] and step times variability on both left [F (1, 73) = 136.66, *p* < 0.001] and right [F (1, 76) = 157.70, *p* < 0.001] legs when walking on the rocky surface compared to the regular surface (Table 4).

### Mediolateral Margin of Stability

For the standard deviation of the MOS of the right leg, an interaction was detected [F (2, 64) = 4.21, *p* < 0.05] and this relationship can be seen in Figure 9B. Overall, active arm swing led to larger values compared to both normal and held arm (*p* < 0.001) strategies when walking on a rocky surface. Also, rocky surface led to larger values compared to regular surface (*p* < 0.001) no matter the arm strategy used (Figure 9B). Statistical analyses showed that rocky surface increased the MOS mean of both the left [F (1, 57) = 99.10, *p* < 0.001] and right [F (1, 56) = 126.49, *p* < 0.001] compared to regular surface for both legs (Table 3). For the SD of this metric on the left leg, active arm swing led to larger values compare to both other arm swing strategies [F (2, 69) = 7.55, *p* < 0.01], while the rocky surface led to increased values [F (1, 46) = 166.70, *p* < 0.001] compared to the regular surface (Table 4).

## Discussion

### Main Findings

This study examined the effects of different arm swing conditions (normal, held and active) on postural control and dynamic stability in healthy young adults when walking on even and rocky surfaces. Our hypotheses were partially supported as overall our results demonstrated that, compared to normal and held arm swing, active arm swing 1) increased trunk kinematics variability and peak values, 2) increased the average step length and step time while increasing step width COV. As for the effect of terrains, results showed 1) increased trunk kinematics variability and peak values as well as larger mean MOS and MOS variability when walking on a rocky surface compared to regular surface, and 2) increased average step width and decreased average step time when walking on rocky compared to regular surface. Finally, rocky surface led to increased COV of all spatiotemporal values (length, width and time).

### Arm Swing

Overall, and in accordance with our hypothesis, walking while actively moving the arms had a destabilizing effect compared to normal or held arm swing. When using the active arm swing strategy, our participants displayed increased mean and peak values of trunk angular velocities in the frontal and transverse planes as well as increased peak values for trunk linear velocities compared to the normal and held arm conditions. The control of the trunk is critical for regulating postural control (Menz et al., 2003; Tucker et al., 2008). Studies using motion capture and inertial sensors placed at the trunk level reported that the increase in linear velocity and angular velocity were indicators of diminished postural control and increased risk of falls (Gill et al., 2001; Goutier et al., 2010; Arvin et al., 2016a; Arvin et al., 2016b; Siragy et al., 2020). This was shown through a variety of tasks, including steady-state gait and obstacle crossing protocols, single-legged and double-legged standing with open and closed eyes (Gill et al., 2001; Goutier et al., 2010; Arvin et al., 2016a; Arvin et al., 2016b; Siragy et al., 2020). In these studies, the more challenging tasks, such as standing with closed-eyes and obstacle gait trials led to higher trunk sway measures, which according to the authors, were indicative of inferior postural stability.

Furthermore, when using the active arm swing, our participants exhibited larger trunk kinematics variability compared to both the normal and held arm conditions. While the COM displacement normally follows a smooth path along the ML and AP directions when walking (Winter, 1995; Tesio and Rota, 2019), the larger trunk kinematics variability found in our results indicated that actively moving the arms disrupted the expected COM’s trajectory. Our results contradicted previous findings by Lulić et al. (2008) and Nakakubo et al. (2014), who both suggested that active arm swing strategy improved trunk stability among young adults (Lulić et al., 2008) and older adults (Nakakubo et al., 2014). However, our findings are in line with Siragy et al. (2020) who found that active arm swing in healthy young adults increases trunk linear and angular velocity variability, which, in turn, reduced the harmonic ratio of the COM in the anteroposterior and mediolateral directions (Siragy et al., 2020). Although the active arm swing led to changes in trunk control in our young healthy adults, this condition was not challenging enough to disrupt the completion of the walking task, nor to cause a fall. However, this could be of importance in fall prone demographics that have a more unstable walking pattern due to their larger trunk angular velocity average and variability (Goutier et al., 2010). In this population, the additional disruption to the COM’s trajectory could increase the difficulty of the task and lead to falls.

Additionally, in response to the larger trunk velocities and variability in the active arm swing condition, participants modified the BOS as indicated by the increase in step length and step width variability (Table 4). This combination could have been used to ensure proper foot placement (Lamoth et al., 2011) in order to maintain adequate levels of stability (Siragy et al., 2020). However, spatiotemporal changes due to active arm swing, may affect the golden ratio (Iosa et al., 2016)**.** Interestingly, despite all the kinematic indicators showing decreased postural control when using the active arm swing strategy, no main effect for arms on average MOS were detected. This indicates that active arm swing did not impact the participants’ global dynamic stability (Table 3). These results suggested that, while active arm swing led to diminished postural control, the foot placement adjustments performed by our participants potentially sufficed to maintain adequate levels of stability (Siragy et al., 2020).

Consistent with our group’s previous works, no significant differences were detected between the normal and held arm swing conditions for all parameters studied (Hill & Nantel, 2019; Siragy et al., 2020). An explanation for this outcome lies within the observations made by Bruijn et al. (2010). When using the held arm strategy, the weight of the arms contributed to the total weight of the trunk which increases trunk inertia. The increased trunk inertia effectively limited the displacement of the COM as well as the trunk linear and angular velocity. This could explain the lack of significant differences in step length, width, and time between the normal and held arm swing strategies as no further spatiotemporal adaptations would have been required. Alternatively, restraining the arms could have triggered a whole-body compensation mechanism to appropriately respond to the changing environment (Marigold, 2002). These compensations included increased activation of the muscles at the trunk and hip levels which could limit the displacement of the COM to maintain levels of stability comparable to those when using a normal arm swing.

### Rocky Surface

Walking on the rocky surface increased the trunk linear velocity and angular velocity averages in the frontal and transverse planes when compared to the regular surface. Furthermore, the rocky surface led to larger peak values for the trunk linear velocity in the frontal plane and angular velocity in all three planes, indicating poorer postural control (Goutier et al., 2010). Finally, this condition resulted in greater variability in trunk linear and angular velocity in the sagittal, frontal, and transverse planes. The oscillations caused by this terrain disrupted the trunk’s intended path (the sinusoidal path described by Winter (1995) and displaced the COM more than the steady-state condition (Brenière and Do, 1991; Gates et al., 2013; Rankin et al., 2014; Winter, 1987). Normally, a healthy gait possesses relatively small amounts of variability (Hausdorff, 2005; Siragy et al., 2020). However, the more variable trunk motion displayed by our participants indicated decreased postural control (Siragy et al., 2020) and most likely explained their more variable foot placement (Table 4) (Winter, 1987; Brenière and Do, 1991; Rankin et al., 2014; Siragy et al., 2020).

Contrary to our hypothesis, walking on the rocky surface led to larger MOS mean and larger MOS SD (Table 3) compared to the regular surface. The larger MOS data showed that the participants were compensating for the perturbation caused by the surface type by modifying their normal gait pattern to maintain stability. This compensation was shown through our spatiotemporal data as the rocky surface walking led to increased step width. Adopting wider steps would increase the BOS within the frontal plane, leading to an increase in the distance between the xCOM and the edge of the BOS (Rosenblatt and Grabiner, 2010). Our spatiotemporal results were consistent with findings by Gates et al. (2013) in participants walking the rocky surface as well as with Onushko et al. (2019) who reported similar spatiotemporal adjustments when participants walked on a surface oscillating in the sagittal and frontal planes. However, it was also shown that walking with wider steps led to more variability in foot placement (Perry and Srinivasan, 2017) and less control over the trunk motion among healthy young adults (McAndrew Young and Dingwell, 2012). This foot placement variability was made evident through the increased coefficient of variation of all spatiotemporal parameters.

Additionally, when walking on the rocky surface, participants also reduced their step time. This could have been done to increase the ratio of double support to single support stance in a challenging environment. Voloshina et al. (2013) also reported decreased step time when walking on an uneven surface. However, as gait speed was controlled, the observed reduction in step length caused the decrease in step time (Voloshina et al., 2013). As our participants walked using a self-paced speed, our observations could have stemmed from a reduction in gait speed when walking on the rocky surface compared to the regular surface. Otherwise, this observation could have been due to the participants feeling an increased risk of falling due to the destabilizing terrain (McAndrew et al., 2010). Indeed, previous research indicates that individuals attempt to traverse destabilizing surfaces quicker, in order to return to a more stable surface, by reducing their step time (Sinitski et al., 2015; Sturk et al., 2019).

### Interaction

Active arm swing did not counteract the negative effects of walking on a rocky surface; it enhanced the destabilizing effect of the rocky surface on the COM displacement. Rocky surface walking led to larger trunk kinematics and spatiotemporal variability than regular surface despite the arm swing strategy used. The combination of active arm swing and rocky surface led to larger values for all parameters showing a significant interaction effect (Figure 9). Consequently, our results did not support our hypothesis that active arm swing would attenuate the effects of the rocky surface on postural control and stability.

There were two exceptions to these findings. First, step time of the left leg was decreased when walking on rocky compared to regular surface. Though, active arm swing still increased this parameter compared to the other arm swing strategies on both regular and rocky surfaces. Lastly, active arm swing reduced trunk angular velocity in the frontal plane when walking on a rocky surface to similar values when walking on a regular surface, showing an improvement in postural control (Goutier et al., 2010; Siragy et al., 2020).

## Limitations

Since active arm swing was shown to benefit stability in steady-state and perturbed walking (Lulić et al., 2008; Nakakubo et al., 2014; Punt et al., 2015; Wu et al., 2016), and our results showed otherwise, these advantages could be situational or based on the perturbation type. Therefore, more research is required to fully comprehend the effect of arm swing when walking. Firstly, participants were secured in a harness throughout the trials. This could have created a sense of confidence and, ultimately, modified the participants’ gait pattern. Secondly, the use of a treadmill may not always be representative of over-ground walking as some use a more “cautious gait” when using a treadmill (Yang and King, 2016). Thus, future research should examine these variables during over-ground walking using a protocol similar to Gates and collaboration (Gates et al., 2012; Gates et al., 2013) where their participants walked on a flat surface and on a surface covered with rocks. Finally, the attentional demands associated to modifying arm swing strategies can affect gait stability. Mofateh et al. (2017) showed an improvement in gait stability as shown through reduced gait spatiotemporal variability while Chow et al. (2019) demonstrated that adopting an internal focus was detrimental to motor performance. Therefore, future studies should ensure maintenance of inter-limb coordination when modifying arm swing.

## Conclusion

In summary, active arm swing and rocky surface walking increased the variability of trunk kinematics and their peak values. Our spatiotemporal data showed that our participants responded to the active arm swing by adjusting their mediolateral foot placement to maintain a pre-existing level of global dynamic stability. In contrast, when walking on the rocky surface, healthy young adults select a foot placement strategy that increases the distance of their COM’s motion state (speed and velocity) to the edge of their BOS in order to increase mediolateral global dynamic stability. The findings of both arm swing and destabilizing surfaces hold several clinical implications. The present findings suggest that the increased arm swing amplitude during our active arm swing condition increases trunk kinematic variability. To reduce the risk of falling, our healthy young adults appropriately adjusted their foot placement to maintain their already existing level of dynamic stability. Therefore, clinicians aiming to reduce future fall risk should consider therapies that facilitate appropriate foot placement adjustment rather than programs that attempt to increase arm swing. This is due to the fact that active arm swing reduces postural control thereby requiring further adjustments by the neuromuscular system to mitigate the risk of falling. This is particularly relevant to fall prone demographics who exhibit a close association between fall risk and reduced or absent arm swing (such as individuals with Parkinson’s Disease). Indeed, attempting to restore arm swing in these individuals, by actively increasing arm swing magnitude, may in fact exacerbate fall risk as additional demands are placed on the neuromuscular system to correctly modify their foot placement. Similarly, protocols that facilitate foot placement adjustment would have direct relevancy in reducing falls on destabilizing surfaces as increasing the base of support, through foot placement adjustment, increases an individual’s mediolateral global dynamic stability.

## Data Availability

The raw data supporting the conclusions of this article will be made available by the authors, without undue reservations.
